# Which experiences of health care delivery matter to service users and why? A critical interpretive synthesis and conceptual map

**DOI:** 10.1258/jhsrp.2011.011029

**Published:** 2012-04

**Authors:** Vikki Entwistle, Danielle Firnigl, Mandy Ryan, Jillian Francis, Philip Kinghorn

**Affiliations:** Social Dimensions of Health Institute, University of Dundee, Dundee; 1 Health Economics Research Unit; 2 Health Services Research Unit, University of Aberdeen, Aberdeen, UK

## Abstract

**Objective:**

Patients' experiences are often treated as health care quality indicators. Our aim was to identify the range of experiences of health care delivery that matter to patients and to produce a conceptual map to facilitate consideration of why they matter.

**Methods:**

Broad-based review and critical interpretive synthesis of research literature on patients' perspectives of health care delivery. We recorded experiences reported by a diverse range of patients on ‘concept cards’, considered why they were important, and explored various ways of organizing them, including internationally recognized health care quality frameworks. We developed a conceptual map that we refined with feedback from stakeholders.

**Results:**

Patients identify many health care experiences as important. Existing health care quality frameworks do not cover them all. Our conceptual map presents a rich array of experiences, including health care relationships (beyond communication) and their implications for people's valued capabilities (e.g. to feel respected, contribute to their care, experience reciprocity). It is organized to reflect our synthesis argument, which links health care delivery to what people are enabled (or not) to feel, be and do. The map highlights the broad implications of the social dynamics of health care delivery. Experiences are labelled from a patient's perspective, rendering the importance of responsiveness to individuals axiomatic.

**Conclusions:**

Our conceptual map identifies and helps explain the importance of diverse experiences of health care delivery. It challenges and helps policy-makers, service providers and researchers to attend to the range of experiences that matter, and to take seriously the need for responsiveness to individuals.

## Introduction

People's experiences of health care – of how they are ‘treated’ in the broader sense as they use health services – have been issues of concern for decades^1–3^ Internationally, various initiatives have been introduced to improve aspects of health care experience such as information provision, choice and dignity but reports of poor experiences continue to emerge and repeated assessments suggest a lack of improvement in several domains.^4,5^

The idea that experiences of health care should be monitored for quality purposes is well established: surveys of patients' experiences are used in many countries to report on and compare service performance, and to inform and evaluate efforts to improve services. However, there are currently many unanswered questions about: which aspects of health care experience matter and why; how well these are reflected in survey instruments and other assessment tools; how they could and should be valued; and how information about them should inform service development.

This paper reports on the first stage of a study commissioned to investigate the development of quantitative estimates of the value of experiences of health care. This first stage aimed: to identify the kinds of experiences that matter to patients; to consider why they matter; and to present them on a conceptual map to support discussions about their assessment and valuation.

## Methods

Our approach was based on critical interpretive synthesis (CIS) (Box 1).^6,7^ We combined a broad-based review of research literature to identify the kinds of experiences of health care that can matter with consideration of *why* these experiences might be important and how they relate to each other. This latter consideration was informed by reflections on conceptual frameworks and theories from health care and from philosophical writings about human experience and wellbeing. We used our critical interpretation of reports of experiences of health care that matter to develop an account of how, in broad terms, different aspects of experience relate to each other, and an explanation of why they can matter. This ‘synthesis argument’^6^ informed the organization of experience concepts on our conceptual map. Draft versions of the map were refined after feedback from workshops with people with diverse expertise and interests in health care.

Box 1 **Key characteristics of critical interpretive synthesis (CIS)^6,7^**

**Table:** 

Purpose	To further understanding of a topic/question by drawing on broadly relevant literature to develop concepts and theories that integrate those concepts. The topic might not be precisely bounded, and the initial question might be refined as the review progresses.
Process	The process of CIS is iterative, interactive, dynamic and recursive, with recognition of a need for flexibility and reflexivity. Searching, sampling, critique and analysis may happen concurrently.
Search strategy	Formal bibliographic searches may feature, but use will also be made of the research team's awareness of relevant literature from various fields and sources. The strategy may evolve organically.
Sampling	Sampling of studies may be selective and purposive (not necessarily aiming for comprehensive identification and inclusion of all relevant literature). Inclusion criteria can be flexible and to some extent emergent. Reflexivity informs sampling. Ongoing selection of potentially relevant literature is informed by emerging theoretical framework.
Quality appraisal	Some formal appraisal of methodological quality may be appropriate, but judgements about the credibility and contribution of studies may be deferred until synthesis, as methodologically weak papers may still prove theoretically or conceptually insightful.
Data analysis	Inductive – aims towards the development of a synthesizing argument. CIS involves an interrogation rather than aggregation of concepts and themes. Formal data extraction may be useful but is not essential to the approach.
Findings/results	CIS results in the generation of a ‘synthesizing argument’ linking existing constructs from the findings to ‘synthetic constructs’ (new constructs generated through synthesis). This network of relationships and categories is submitted to rigorous scrutiny as the review progresses.
Discussion, contribution	CIS aims to offer a theoretically sound and useful account that has explanatory power and is demonstrably grounded in the evidence. It explicitly acknowledges the ‘authorial voice’ and that some aspects of its production will not be auditable or reproducible.

### Review scope

We focused on research into experiences of the way health care is provided as contrasted with research into experiences of health conditions or experiences of particular health care technologies and their effects and side-effects – although we recognize that (subjective) experiences of these are inter-related.

We acknowledged multiple meanings of the word ‘experience’ and decided to attend to both reports of health care *events* that people were involved in and reports of *how people were affected* by these events, including what they thought and felt about them at the time and subsequently. We judged that, although people from different social backgrounds and people using different types of health service would vary in terms of what they experienced and how much importance they attached to particular kinds of experience, there would be enough commonality to justify attempting to develop a generic conceptual map that could serve as a starting point for discussion of experiences across a range of health care settings in high income countries.

### Review sources and sampling

Health care experiences are recounted and evaluative judgements of experiences expressed in many social settings using various communication genres and media. We worked primarily from peer-reviewed research reports because: research into patients' perspectives has proliferated in recent decades and we were confident we could generate a sample of reports that would give insight into the views of diverse users of a range of health services; bibliographic indexing and abstracts could facilitate the identification and initial appraisal of studies; reports usually summarise the experiences and views of people who have been carefully sampled and described; and they often note critical considerations relating to the interpretation of findings.

Search strategies were developed for Medline, EMBASE, Psycinfo and ASSIA (up to 2009) to identify studies reporting patients' experiences of health care delivery. Sensitivity was prioritized over specificity. Three researchers used a multi-step process to develop a sample of studies. We sought to construct a maximum variation sample^8^ that was diverse in terms of the types of health service (e.g. maternity, palliative care) and the people (e.g. children, ethnic minorities, people living in economically disadvantaged communities) involved. Data extraction commenced before the sampling of studies was complete, so we could also ensure we considered diverse domains of health care experience.

Consistent with CIS, when sampling papers, we were driven more by conceptual relevance than methodological rigour.^6^ We sought to maximize the range of experiences identified as important rather than to accumulate multiple mentions of similar concepts or to estimate how much particular experiences matter.^7^ As the review progressed, we prioritized in-depth qualitative research and reviews that provided experiential insights not previously recorded for the review. We stopped sampling when we had covered the available range of services and patients and data extraction was no longer identifying substantially new experience concepts.

### Data extraction and interpretation and grouping of concepts

For each study included in our sample, we extracted basic information about study aims and methods, and prepared ‘concept cards’ to record the kinds of health care experience identified as mattering. We took a broad view of what counted as mattering, attending not only to experiences that patients explicitly designated important or associated with health care quality but also to experiences they highlighted as particularly good or bad, or otherwise (implicitly) deemed worthy of comment. We focused on patients' rather than service providers' perspectives, and extracted information about diverse or conflicting views where these were reported. The concept cards included fragments of quotations from study participants, thematic labels assigned by researchers and/or summarizing phrases of our own.

We considered various conceptual and theoretical frameworks that might guide our thinking about *why* experiences of health care can matter, and about how they might be organized on a conceptual map. We looked initially at the World Health Organization's ‘Responsiveness framework’,^9^ the Institute of Medicine's domains of health care quality,^2^ and Nolan *et al.*'s ‘SENSES’ framework (Box 2).^10^ Focusing on one framework at a time, we took a diverse sample of concept cards and attempted to place each of them under the framework headings. We discussed and noted our reasons for thinking whether and how well particular concepts fitted under the headings.

Box 2 **Domains of three widely used health care quality frameworks**

**Table:** 

**World Health Organization: responsiveness** *Health care systems ensure:*	
Autonomy	(Of patient/family) via provision of information about health status, risks and treatment options; involvement of individual/family in decision-making if they want this; obtaining of informed consent; existence of rights to treatment refusal
Choice	Of health care providers
Clarity of communication	Providers explain illness and treatment options, patients have time to understand and ask questions
Confidentiality	Of personal information
Dignity	Care is provided in respectful, caring, non-discriminatory settings
Prompt attention	Care is provided readily or as soon as necessary
Quality of basic amenities	Physical infrastructure of healthcare facilities is welcoming and pleasant
Access to family and community support	(For hospital inpatients)
**Institute of Medicine: quality of care** *Health services are:*	
Safe	Avoiding injuries to patients from the care that is intended to help them
Effective	Providing services based on scientific knowledge… avoiding underuse and overuse
Patient-centred	Providing care that is respectful of, and responsive to individual patient preferences, needs and values, and ensuring that patient values guide all clinical decisions
Timely	Reducing waits and sometimes harmful delays
Efficient	Avoiding waste, including of equipment, supplies, ideas, energy
Equitable	Providing care that does not vary in quality because of personal characteristics
**Nolan *et al*: SENSES framework** *All parties should experience relationships that provide a sense of:*	
Security	To feel safe within relationships
Belonging	To feel part of things
Continuity	To experience links and consistency
Purpose	To have potentially valuable goal(s)
Achievement	To make progress towards desired goal(s)
Significance	To feel that you matter

Our interpretive reflections on the extracted experience concepts, informed by our wider reading, led us to develop a synthesis argument^6^ that could, in a general sense, describe relationships between different types of experience concepts and help explain why diverse experiences of health care can matter. We used the synthesis argument to help structure our conceptual map.

We organized two workshops with patient advocates, government policy officers, and clinical and academic researchers to test draft versions of our conceptual map. Participants agreed to serve as critical friends. At the start of the workshops, we asked them to think about individual experiences of health care use (their own or others') and what had mattered about these. We then described the development and key features of our conceptual map, and asked participants to try to locate these individual experiences and examples of experiences summarized on concept cards, on it. Participants gave constructive critical feedback about the map in discussion and by annotating draft copies.

We continued to check the map's coverage of experience concepts from research studies not included in our primary sample and invited critical comments from additional stakeholder representatives after further presentations. We did not need to make substantial changes after the post-workshop modifications.

## Results

### Experiences identified

The bibliographic searches identified 9,224 unique references. Relatively few were relevant because our search strategy prioritized sensitivity. We retrieved full text papers for 190 potentially eligible studies, and included 77 studies in the primary source sample used to generate concept cards.

The source studies reported on diverse experiences of health care delivery, often generating over 20 concept cards each. We noted early in the review that patients' experiences could be considered in two categories. First, what health services and (especially) staff are like and what they do. Patients comment on how systems seem to work and how staff behave, particularly in terms of relating and responding to patients and family members. Usually more tentatively, they also make inferences about the characteristics of services and staff (including resource levels, skills, and motivations) that underlie or ‘cause’ the behaviours they see. Second, how patients or family members feel as a result of what services and staff are like and do, and what they consider themselves enabled (or not) to be and do.

### Conceptual frameworks

We could not place all the concept cards we considered under the headings suggested by the three frameworks. In part this was because the frameworks focused *either* on what health services and staff are like and what they do^2,9^ *or* on how patients feel in health care relationships,^10^ but not both. However, even if we limited the selection of concept cards to the broad category that most suited the framework, not all could be accommodated without distorting the definitions supplied with the frameworks and/or developing speculative (often tenuous) links to connect the experience concepts to the framework headings. Box 3 illustrates how some concept cards (column 1) might be placed (columns 2–4). It highlights some gaps in particular frameworks, and shows how some framework headings, such as patient centred care, are accommodating but at the cost of obscuring potentially important distinctions.^11–18^

Box 3 **Selected examples of experiences noted on concept cards and possible allocations to existing framework headings**

**Table:** 

Example concept cards	WHO responsiveness	IOM quality	Nolan ***et al*. SENSES**
***a) Focusing on what health services and staff do:***			
Nurses demonstrate calm confidence when diffusing potentially violent disturbances on the ward *(inpatient mental health)*^15^	?	Safe	[Security]
Clinicians are gentle when conducting investigations *(childhood asthma)*^13^	Dignity	Patient-centred	[Security]
The doctor does not understand the sort of life people [in this deprived area] are really living *(general practice)*^11^	?	Patient-centred	? [Continuity]
Staff are paternalistic and coercive *(mental health)*^15^	Autonomy	Patient-centred	? [Significance]
***b) Focusing on how service users feel or are positioned:***			
‘I always felt my opinion counted for something’ *(maternity services)*^14^	Autonomy Dignity	Patient-centred	Belonging Significance
‘You feel as if you're taking up the doctor's time… That makes you feel under pressure’ *(general practice)*^11^	? [Dignity]	? [Patient-centred]	? [Security] [Significance]
Patients feel apprehensive in the presence of other patients (in a mixed sex pre-operative waiting area) *(day case surgery)*^16^	? [Dignity]	[Safe] [Patient-centred]	Security
‘feel respected as part of the team, fighting the same battle’ *(breast cancer)*^17^	Dignity ?	[Patient-centred]	Belonging Significance Purpose
‘I didn't even know there was such a thing as palliative care’ *(cancer palliative care)*^12^	[Autonomy] Clarity of communication	?	?
***c) Linking what health services and staff do with how service users feel or are positioned:***			
Health professionals enable parents to fulfil their roles as carers and brokers of information to their children *(childhood asthma)*^13^	Autonomy Access to family	Patient-centred	Belonging Continuity Purpose Achievement
‘The nurse used to come and tell me who she was for the shift. I have bad eyesight, so it really helped me. It showed she cared and it helped me to talk to her if I needed’ *(inpatient mental health)*^15^	Dignity, clarity of communication	Patient-centred	[Security] [Significance]
[Woman recounts asking what her blood pressure was, being told ‘It's OK’, asking for numbers, being told she didn't need to know]. ‘Then I felt like I was being combative’ *(maternity services)*^14^	Autonomy, clarity of communication, dignity ?	Patient-centred	[Significance] [Belonging] [Security]
Staff lack enthusiasm, patients feel undeserving of care *(mental health)*^15^	?[Prompt attention]	? [Patient-centred]	? Significance
WHO = World Health Organization; IOM = Institute of Medicine			

Given this lack of fit, we developed our conceptual map to facilitate discussion of experiences of health care delivery. We kept in mind the need to address the question of *why* the experiences that people commented on might be important. We attended to reasons given by study participants and authors, and noted that participants' experiences of how they felt and who and what they were enabled to be and do often featured as reasons why it mattered what services and staff were like and did. We asked ourselves repeatedly, why is that important, and recognized that social and relational aspects of health care delivery can have important implications for the identities and self-evaluations, as well as the behaviours, that patients can adopt.^19–21^ We based our emerging interpretive synthesis argument on the idea that health care delivery affects the quality of people's lives.

Considerations of the quality of people's lives focus variously on happiness, desire or preference satisfaction, broad subjective wellbeing including life satisfaction, and opportunities or functionings that are normatively deemed good.^22,23^ Some support for each of these could be derived from patients' accounts but we chose to reflect the capabilities approach associated particularly with Sen,^24^ in our synthesis argument. The capabilities approach considers the genuine opportunities that people have to be and do what they value. It is particularly well suited to reflecting the importance of people's experiences of being respected (or not) as individuals with an interest in their own particular identities and lives, and of being enabled (or not) to engage with their health care providers and in their health care in ways that they value, and have reason to value.

**Figure 1:**
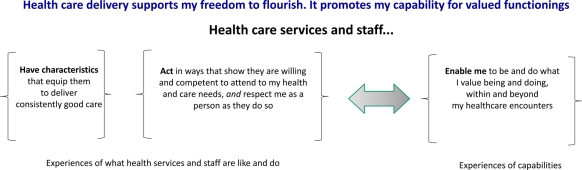
Basic structure of conceptual map

The synthesis argument, which is reflected in the headings used to organize our conceptual map (Figure 1) can be summarized as:

The characteristics and actions of health care services and staff, and the ways they relate to patients, have implications for patients' experiences of being enabled (or not) to feel, be and do what they value feeling, being and doing – in the course of their health care contacts and beyond. Experiences of health care delivery matter because they shape and represent capabilities that are key to how well people's lives can go.

### Decisions and processes in map development

**Figure 2:**
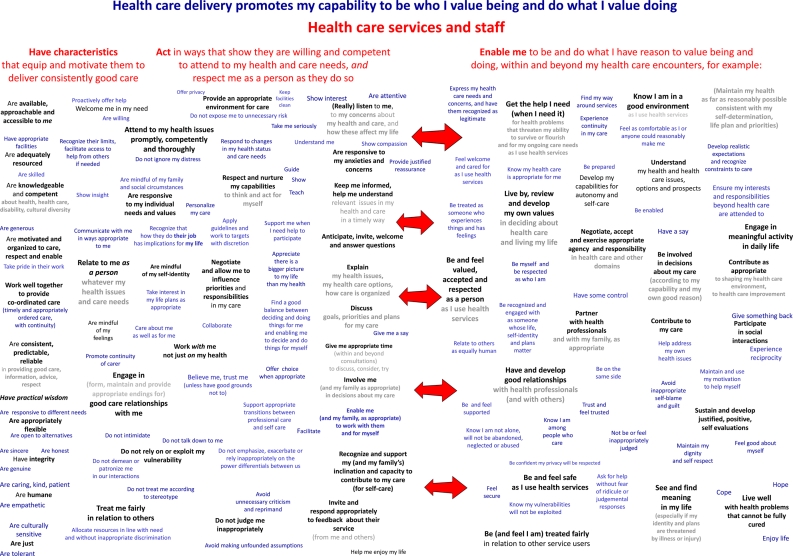
Conceptual map of experiences of health care delivery

We organized the experiences presented on the conceptual map (Figure 2) into three main groups under the headings (in red) ‘Health care services and staff’: ‘Have characteristics that equip them to deliver consistently good care’; ‘act in ways that show they are willing and competent to attend to my health and care needs, and respect me as a person as they do so’; and ‘enable me to be and do what I value being and doing within and beyond my health care encounters’.

We decided to present *both* experiences in terms of what health services and staff are like and do *and* experiences in terms of how patients feel and are enabled, for several reasons. First, although patients' feelings and capabilities are more obviously akin to outcomes or endpoint evaluation criteria, they are complex and dynamic products of what both patients and providers bring to encounters, so cannot be solely attributed to features of health care delivery. For example (using concept labels from the map to illustrate), some people ‘develop [significant] capabilities for autonomy and self care’ and ‘contribute to [their] care’ *despite* receiving care from staff who *fail* to respect and nurture [these] capabilities'. Similarly, staff might welcome a person into their service, ‘attend to [their] health issues promptly, competently and thoroughly’, ‘care [genuinely] about [them]’ and strive to be ‘responsive to [their] individual needs and values’, but the person's past history of being treated disrespectfully and abused might preclude their ‘[having] good relationships with health professionals’ or ‘feel[ing] safe as [they] use health services’. Second, attention to structure and process aspects of health care delivery can be important for efforts to improve health care experiences in terms of how patients feel and are enabled.^24,25^

The experiences of what health services and staff are like and do range from underlying characteristics (on the far left) through forms of action described in ways that reflect broad principles and goals (in the middle) to more specific – mainly communicative – behaviours that can help achieve these (immediately to the left of the arrows).

We excluded the underlying characteristics of health services and staff from early versions of the conceptual map because patients are not usually well placed to judge them accurately. However, research team members and workshop participants were concerned about the absence of structural aspects of health care quality.^25^ On further reflection, we decided to represent them on the map because even if they are not well-founded, patients' beliefs about experiences of them can have important implications for their subjective experiences and broader capabilities. For example, a belief that a nurse lacked a genuine commitment to care could tend to undermine a person's ability to ‘feel safe’, ‘valued’ and ‘respected’ and might preclude a person from speaking up about concerns about their care.^20^Attention to the personal and service characteristics that underlie important behaviours might also be important for quality improvement, and some features of staff behaviour can only be considered over multiple encounters (e.g. ‘consistent, predictable, reliable [provision of] care’).

The main concept labels (in black in Figure 2) below the headings represent the key experiences identified from the review. Near-synonyms and examples (in blue) are intended to help clarify meaning without over-defining key concepts. We had to make many judgements about how to group and name concepts, and we recognize that good alternatives are possible. To ensure broad relevance, we used generic labels (e.g. staff rather than doctors, nurses *etc*.) and aggregated the specific experiences noted in source studies up to levels where a broad consensus seemed likely (e.g. ‘communicate in ways appropriate to me’ rather than ‘call me Mrs X instead of by my first name’ or ‘accommodate my sight impairment’). We aimed to convey a reasonably clear sense of the main types of health care experience identified without over-specifying them.

We wanted the map to present features of health care experience to be aspired to, so we phrased most concepts positively. The few that start ‘[health care staff] do not….’ represent experiences that were only mentioned in negative forms in source studies – possibly because they relate to strong norms of behaviour that people do not think to comment on unless they are breached.^26^

To encourage consideration of the perspectives of patients, we worded the concepts to position the reader as a patient. We used double-headed arrows to indicate that the characteristics and actions of health care services and staff impact on what patients feel and how they are enabled, and that patients' capabilities can influence staff actions. The arrows are placed generally because the many plausible and important links between particular concepts on the left and right of the map are not simple, linear and consistent - and an attempt to illustrate all plausible links would look like a plate of spaghetti!

The conceptual map is messy even without spaghetti arrows, but our attempts to reduce the number of concepts and/or impose further structure by grouping concepts more hierarchically were unsatisfactory: they lost too much of what we wanted to portray, and precluded or obscured other potentially useful arrangements. Although higher level groupings will be necessary for some purposes, we present the full messy version here because it better reflects the diversity of experiences patients considered important and the complexity of the issues and concerns at stake.^27^ It also leaves more possibilities open for future groupings based on this work.

### Conceptual map testing and refining

It was clear at workshops that people need time to become familiar with the conceptual map. In response to workshop participants' suggestions, we clarified headings, added concepts relating to service characteristics (see above), and increased the explicit coverage of some kinds of experience by expanding concept labels or adding more illustrative examples. We have made further minor revisions in response to our own checks of how well the map accommodates insights from studies not included in our original sample.

## Discussion

A broad-based literature review identified a range of experiences of health care delivery that can matter to patients. These experiences were not all reflected in existing frameworks for considering health care quality.

We drew on the capabilities approach to develop a synthesis argument that explains how the various experiences of health care delivery are related, and why they are important. It links the characteristics and actions of health care services and staff to patients' experiences of being enabled (or not) to feel, be and do what they value feeling, being and doing – during health care contacts and beyond.

Our new conceptual map includes a rich array of experience concepts, organized to reflect the synthesis argument. The range of the experiences covered, the way experiences are organized, and the way experience labels are worded have significant implications for the way the map can contribute to efforts to improve experiences of health care delivery. We briefly consider these implications before outlining how policy-makers, service providers and researchers might use the map.

### Implications of map features

#### Range of experiences covered

Our conceptual map attends to experiences of what health services are like and do, and of the impact of health care delivery on people's feelings and capabilities. Compared to existing health care quality frameworks,^2,9^ our map is more explicit and comprehensive in its coverage of the features and implications of interpersonal relationships between health service staff and users. For example, it considers not only communication to support understanding of health issues and treatment choices, but also attitudes and positioning within relationships, and the implications of these for patients' capabilities, including individual identities, self-evaluations and capabilities. The conceptual map promotes expansive thinking about health care experiences which we believe is warranted. The concepts were derived from a review of what matters to patients and many are recognised as morally salient (even associated with human rights) in domains beyond health care.^24^

#### Organization of concepts

The conceptual map is organized to reflect the synthesizing argument developed from the review, and so makes a case as to why the experiences it presents matter. Although a static two-dimensional map cannot adequately reflect the dynamic nature of relationships between experience concepts, the use of generally placed double headed arrows indicates the complex interplay between multiple features of health care delivery and the felt experiences and situated capabilities of patients. We leave it open to users of the map to explore and pick from the many possible paths that can be taken across it in either direction.

#### Phrasing of concepts

The level of abstraction of concepts helps ensure that the map can reflect what matters to diverse patients. Perhaps more significantly, the decision to present the concepts on the map from the perspective of a patient renders the importance of the responsiveness of service provision to individual patients axiomatic. To say that they have acted in accordance with concepts on the left hand side, staff must ensure they have attended to particular individuals and their specific situations as such. Highly standardized service procedures and staff behaviours will not secure for all patients the capability experiences on the right hand side of the map.

The descriptions of experiences are presented positively for aspirational reasons, but negative descriptions of experiences will often be appropriate when summarizing experiences in practice and identifying scope for service improvement.

### Potential uses of the conceptual map

The conceptual map might be used by policy-makers, service providers, researchers and patient advocates as a valuable resource to:

- Help make a case for improving social and relational aspects of health care delivery and patients' experiences. It can help illustrate why these matter;
- Check at a general level that all relevant domains of health care experience have been covered in policy, practice or research initiatives. It can help identify potential gaps, for example, in questionnaires used to monitor experiences of health care delivery;
- Stimulate the identification and/or analysis at a more detailed level of experiences of health care delivery that might warrant attention in particular situations. It can prompt mention and investigation of specific examples of reported experience;
- Provide a reminder of the dynamic nature of the production of patient experiences and discourage inappropriate reductionism in the provision and assessment of health care;
- Challenge and help people to take seriously the idea that health care delivery should be responsive to individual patients. It illustrates, for example, that responsiveness requires much more than offering choice, and highlights the problems of thinking about health care experiences in terms of simple checklists of staff behaviours.

#### Future developments

There is scope to improve the conceptual map. Although we sampled source documents carefully from a broad literature review, and the map has been carefully reviewed by a range of key stakeholders, other research teams would not have selected the same studies, extracted the same concept cards or used the same words and spatial arrangements to represent health care experiences that can matter. The map is intended to start a discussion, not as a fixed and ideal representation of what matters most in all health services and for all people. We think it is broadly applicable across a range of people and health care settings but we recognize that some terms will not appeal to all. We welcome participatory efforts to modify the map for particular applications, although we encourage retention of the basic structure (reflecting the synthesis argument) and the single patient perspective (to preserve the commitment to individual responsiveness). The use of the map in practice has yet to be evaluated, but may be enhanced by the development of supporting resources.
